# Incorporation of Functional Rubisco Activases into Engineered Carboxysomes to Enhance Carbon Fixation

**DOI:** 10.1021/acssynbio.1c00311

**Published:** 2021-10-19

**Authors:** Taiyu Chen, Yi Fang, Qiuyao Jiang, Gregory F. Dykes, Yongjun Lin, G. Dean Price, Benedict M. Long, Lu-Ning Liu

**Affiliations:** † Institute of Systems, Molecular and Integrative Biology, University of Liverpool, Crown Street, Liverpool L69 7ZB, U.K.; ‡ National Key Laboratory of Crop Genetic Improvement and National Center of Plant Gene Research, Huazhong Agricultural University, Wuhan 430070, China; § Australian Research Council Centre of Excellence for Translational Photosynthesis, Research School of Biology, Australian National University, 134 Linnaeus Way, Acton, Australian Capital Territory 2601, Australia; ∥ College of Marine Life Sciences, and Frontiers Science Center for Deep Ocean Multispheres and Earth System, Ocean University of China, Qingdao 266003, China

**Keywords:** bacterial microcompartment, carboxysome, CO_2_ fixation, CO_2_-concentrating mechanisms, Rubisco, *Rubisco activase*

## Abstract

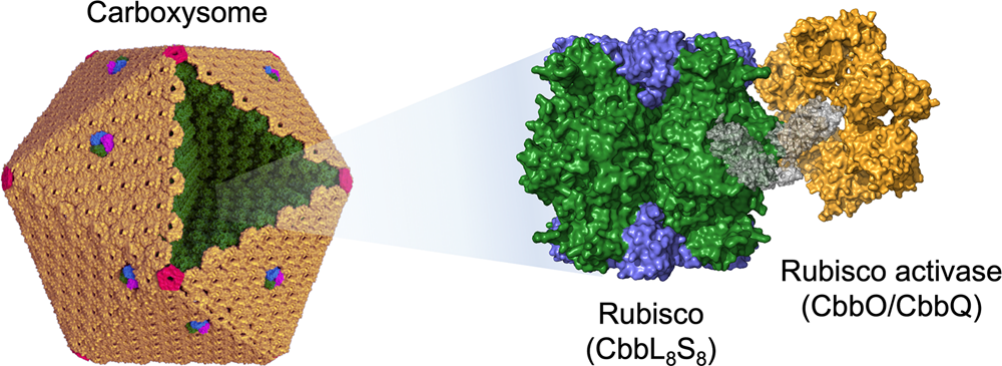

The carboxysome is a versatile paradigm of prokaryotic organelles and is a proteinaceous self-assembling microcompartment that plays essential roles in carbon fixation in all cyanobacteria and some chemoautotrophs. The carboxysome encapsulates the central CO_2_-fixing enzyme, ribulose-1,5-bisphosphate carboxylase/oxygenase (Rubisco), using a polyhedral protein shell that is selectively permeable to specific metabolites in favor of Rubisco carboxylation. There is tremendous interest in repurposing carboxysomes to boost carbon fixation in heterologous organisms. Here, we develop the design and engineering of α-carboxysomes by coexpressing the Rubisco activase components CbbQ and CbbO with α-carboxysomes in *Escherichia coli*. Our results show that CbbQ and CbbO could assemble into the reconstituted α-carboxysome as intrinsic components. Incorporation of both CbbQ and CbbO within the carboxysome promotes activation of Rubisco and enhances the CO_2_-fixation activities of recombinant carboxysomes. We also show that the structural composition of these carboxysomes could be modified in different expression systems, representing the plasticity of the carboxysome architecture. In translational terms, our study informs strategies for engineering and modulating carboxysomes in diverse biotechnological applications.

## Introduction

Cells exploit the physical and chemical nature of molecules to generate self-assembling supramolecular complexes, membrane domains, and organelles, which provides a means for segregating specific functions into different subcellular regions to modulate metabolic reactions in space and in time.^1,2^ While the emergence of compartmentalization and confinement in the cell is widely accepted as a key event in the evolution of eukaryotic cells, more recent work has documented that compartmentalization is also ubiquitous in prokaryotes. A versatile paradigm is the bacterial microcompartment (BMC) that encapsulates diverse metabolic enzymes within the nanoscale compartments using a polyhedral protein shell.^3−9^ BMCs are widespread in the bacterial phyla and are of paramount importance for CO_2_ fixation, pathogenesis, and microbial ecology.^10−12^

**Figure 1:**
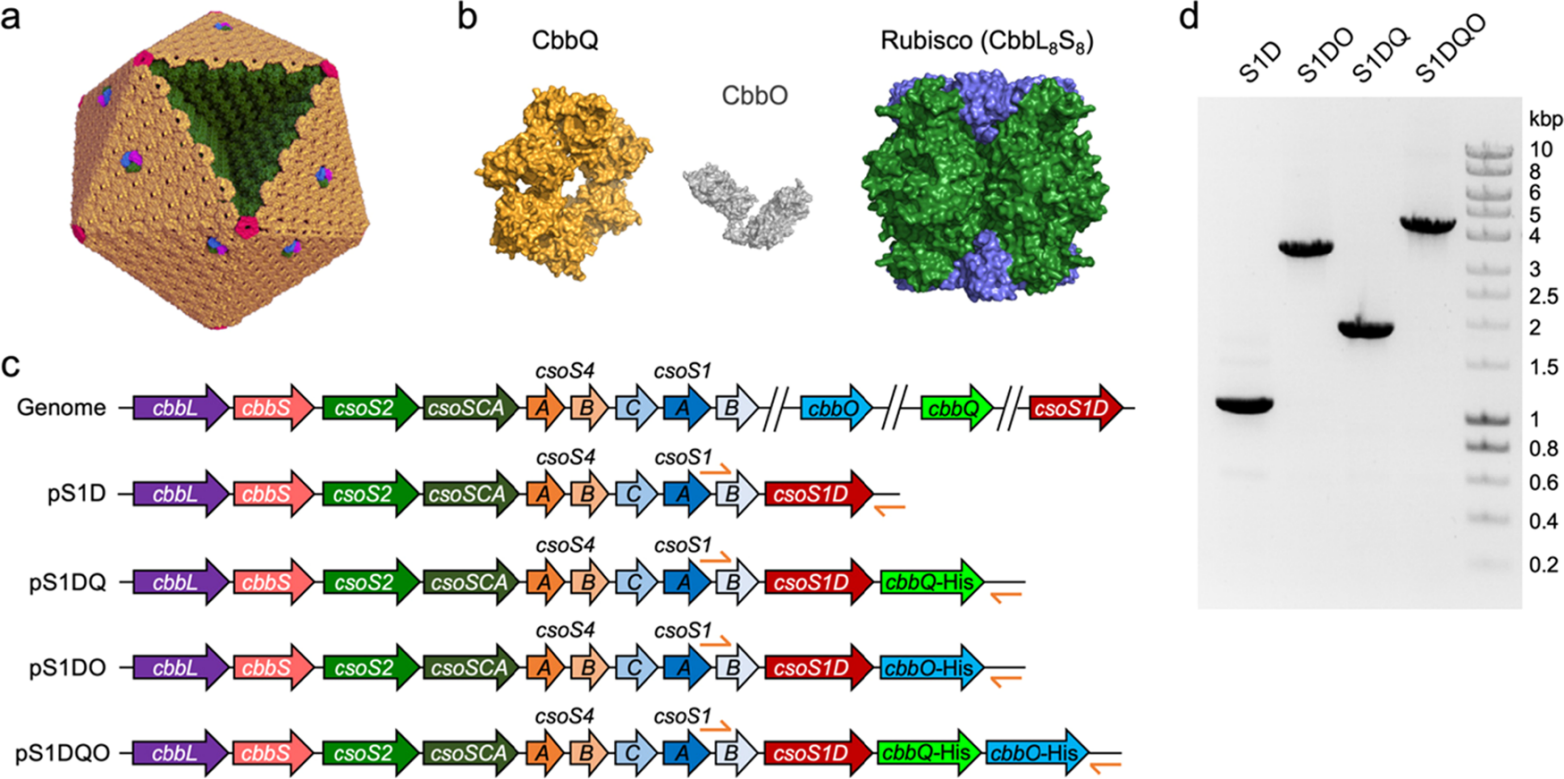
Strategies for incorporating CbbQ and CbbO into recombinant α-carboxysomes. (a) Schematic model of the icosahedral α-carboxysome structure. Rubisco (CbbL_8_S_8_) and carbonic anhydrases (CsoSCA) are enclosed within a semi-permeable shell, which is composed of hexamers (CsoS1A/B/C, yellow), pentamers (CsoS4AB, red), and trimers or pseudohexamers (CsoS1D, purple–blue–green). (b) Model of the association of the Rubisco activase CbbQ hexamer and the adaptor protein CbbO with Form 1A Rubisco (PDB ID: CbbQ, 3ZW6; Rubisco, 1SVD; CbbO, hypothetic structure predicted by I-TASSER server). (c) Genetic organizations of the native α-carboxysome operon in the genome of *H. neapolitanus* and the synthetic operons for producing α-carboxysome structures in *E. coli*. pS1D, pS1DQ, pS1DO, and pS1DQO were generated using a pAM2991 vector. His-tags are fused to the 3′’ end of *cbbQ* and *cbbO* genes. (d) PCR (polymerase chain reaction) verification of the carboxysome-expressing vectors using the primers shown in (c) (Table 3). The sizes of the PCR products were 1.1, 3.5, 1.9, and 4.4 kb for pS1D, pS1DO, pS1DQ, and pS1DQO, respectively.

Carboxysomes are the canonical BMCs found in all cyanobacteria and some chemoautotrophs. Carboxysomes encapsulate the key CO_2_-fixing enzymes ribulose-1,5-bisphosphate carboxylase oxygenase (Rubisco) and carbonic anhydrase (CA), using a protein shell made of numerous protein paralogs (Figure 1a).^8,13,14^ Rubisco is the central enzyme in the Calvin–Benson–Bassham cycle of photosynthesis, mediating CO_2_ fixation by catalyzing the carboxylation of its substrate ribulose-1,5-bisphosphate (RuBP). Although Rubisco is highly productive on a global scale, collectively fixing about 10^11^ tons of carbon annually,^15^ this enzyme is somewhat inefficient given its distinct substrate specificity for both CO_2_ and O_2_ and relatively slow catalytic rate. These features make the catalytical reaction of Rubisco the limiting step in photosynthetic CO_2_ fixation.^16^ To overcome this, in the carboxysome-containing organisms, Rubisco is encased by a protein shell that is selectively permeable to HCO_3_^–^, permitting substantial accumulation of HCO_3_^–^ within the organelle.^17^ The coencapsulated CA then dehydrates HCO_3_^–^ to CO_2_ and supplies a high concentration of CO_2_ around Rubisco.^18,19^ The exquisite carboxysome architecture and the semipermeability of the protein shell ensure enhanced CO_2_ assimilation capacity of carboxysomes that are estimated to contribute to approximately 25% of global carbon fixation.^8^ Introducing functional carboxysomes into heterologous organisms via synthetic biology approaches has proven to be a promising strategy to supercharge CO_2_ fixation and enhance agricultural productivity.^20−26^

Based on the types of the enclosed Rubisco, carboxysomes can be categorized into α-carboxysomes that contain Form 1A Rubisco and β-carboxysomes that encase plantlike Form 1B Rubisco.^8,27^ Rubisco of the two forms is a hexadecameric complex composed of eight large subunits and eight small subunits, denoted as CbbL_8_S_8_ in α-carboxysomes or RbcL_8_S_8_ in β-carboxysomes. The biogenesis of Rubisco requires a series of chaperones, such as GroELS,^28^ Rubisco assembly factor 1 (Raf1),^29−31^ and RbcX^32,33^ for Form 1B Rubisco. Rubisco also requires conformational repair by Rubisco activases (Rca) to be catalytically active. To fulfill the functionality, the active site of Rubisco must be carbamylated by nonsubstrate CO_2_ molecules. However, binding of RuBP prior to carbamylation or other misfire sugar bisphosphates, such as xylulose-1,5-bisphosphate, 2,3-pentodiulose-1,5-bisphosphate, and 2-carboxy-d-arabinitol-1-phosphate, can inhibit Rubisco by closing the catalytic site and impeding reactions with either CO_2_ or O_2_.^34^ Rca is required to remove these inhibitors from Rubisco to restore its carboxylation activity,^35^ through binding with Rubisco over one of the catalytic sites of red-type Rubisco^36^ or the RbcL N-terminus of Form 1B Rubisco.^37^

In the chemoautotroph *Halothiobacillus neapolitanus* (*H. neapolitanus*), Rca comprises a prokaryotic AAA+ protein CbbQ (∼30 kDa) and a Rubisco adaptor CbbO (∼82 to 88 kDa) (Figure 1b).^38,39^ CbbQ appears as a hexameric ring of the typical AAA^+^-ATPase domain and was indicated to be associated with the α-carboxysome by interacting with the shell protein CsoS1.^39^ CbbO has a C-terminal VWA domain with a metal ion-dependent adhesion site, which is vital for interacting with Rubisco.^35,38^ Both *cbbQ* and *cbbO* genes are often present concurrently downstream of the Rubisco genes in the carboxysome-encoding operons.^40^ It has been shown that one CbbQ hexamer can bind one CbbO monomer in vitro to form a bipartite complex, and the binding of CbbO was presumed to be key for the Rca activity.^38^ While evidence indicates that CbbQ is associated with the *H. neapolitanus* carboxysome shell,^39^ how the CbbQO complex promotes activation of Rubisco in α-carboxysomes remains enigmatic.

Here, we develop genetic constructs to coexpress the CbbQO Rca complex with the *H. neapolitanus* α-carboxysomes in *E. coli*, and characterize the incorporation of CbbQO within the recombinant carboxysomes and their roles in promoting CO_2_ fixation of the carboxysomes. Our study provides insight into the significance of Rca in mediating the structure and functionality of α-carboxysomes. It has implications for synthetically engineering carboxysome structures with the capacity of modulating their composition and functionality.

## Results and Discussion

### Integration of CbbO and CbbQ in the α-Carboxysome

Previous studies have shown that expressing the *H. neapolitanus* α-carboxysome *cso* operon could lead to the formation of catalytically functional α-carboxysome structures in *E. coli*(20,21,26,41) and a Gram-positive bacterium.^22^ To coexpress CbbO and CbbQ with recombinant α-carboxysomes and investigate their functions in carboxysome activities, we generated a series of constructs using a pAM2991 vector (Figure 1c). The pS1D plasmid consists of the α-carboxysome *cso* operon from *H. neapolitanus*, including the genes encoding Rubisco large and small subunit proteins (CbbL and CbbS), the shell proteins CsoS1A/B/C and CsoS4A/B, the shell-associated protein CsoS2, the CA protein CsoSCA, and the *csoS1D* gene. The pS1DQ, pS1DO, and pS1DQO plasmids integrate the *cbbQ*, *cbbO*, and *cbbQO* genes, respectively, into the α-carboxysome expression operon, downstream of *csoS1D* (Figure 1c,d). Polyhistidine tags were fused to the C-termini of CbbQ and CbbO for immunoblot assays.

**Figure 2:**
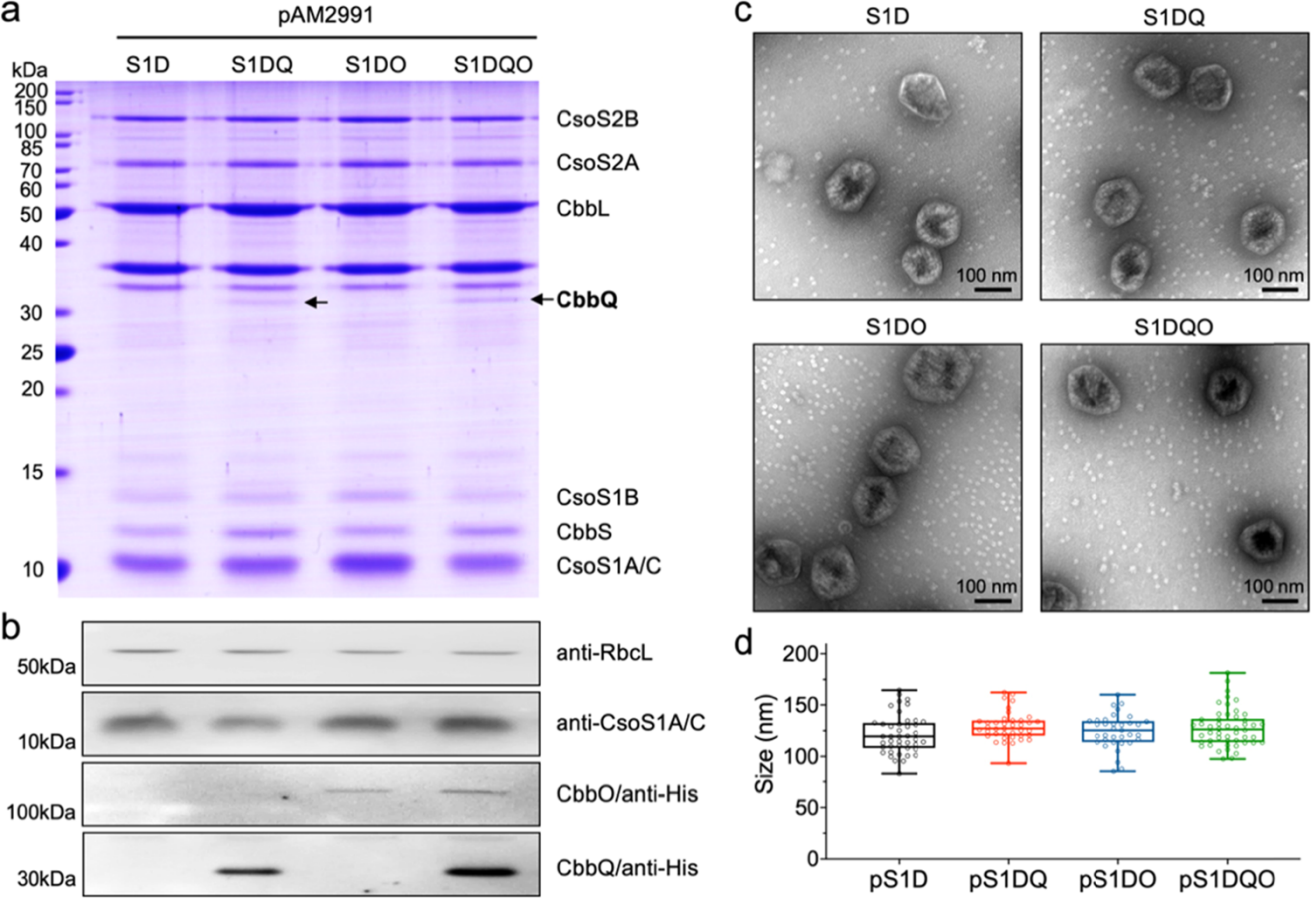
Expression, purification, and immunoblot analysis of the recombinant α-carboxysomes. (a) SDS-PAGE reveals the main protein components of isolated recombinant α-carboxysomes. The carboxysome proteins were annotated based on their molecular weights and immunoblot results. The bands between CbbL and CbbQ are two membrane proteins from *E. coli.* (b) Immunoblot analysis of isolated α-carboxysomes using anti-RbcL, anti-CsoS1, and anti-HisTag (for CbbO and CbbQ) antibodies, suggesting the expression profiles of CbbL, CsoS1, CbbQ, and CbbO in different α-carboxysome structures. (c) Electron microscopy (EM) images of isolated recombinant α-carboxysomes. (d) Diameters of isolated α-carboxysomes measured based on the EM images: 122 ± 19 nm for pS1D (*n* = 43), 129 ± 14 nm for pS1DQ (*n* = 39), 124 ± 17 nm for pS1DO (*n* = 35), and 128 ± 18 nm for pS1DQO (*n* = 50). Data are presented as mean ± standard deviation (SD).

After isopropyl β-d-1-thiogalactopyranoside (IPTG) induction to ensure the expression of α-carboxysome proteins, the recombinant α-carboxysomes were purified by sucrose gradient centrifugation. Sodium dodecyl sulfate polyacrylamide gel electrophoresis (SDS-PAGE) and immunoblot analysis confirmed the presence of the carboxysome protein components in the carboxysome preparations from pS1D, pS1DQ, pS1DO, and pS1DQO cells (Figure 2a,b), consistent with previous results.^41^ In addition, we verified the presence of CbbQ in the pS1DQ and pS1DQO carboxysomes and the incorporation of CbbO into the pS1DO or pS1DQO carboxysomes using an anti-His antibody (Figure 2a,b), demonstrating that CbbQ and CbbO can be structurally integrated into recombinant α-carboxysomes as intrinsic components. Consistently, CbbQ has been identified in the *H. neapolitanus* α-carboxysomes.^39^

Quantitative analysis of immunoblots indicated that the ratio of CbbQ and CbbO in the pS1DQO carboxysomes is ∼6:1 (data were calculated from immunoblot results in Figure 2b), supporting the functional forms of CbbQ as a hexamer and CbbO as a monomer.^38^ Our results suggest that the expressed CbbQ or CbbO alone can be integrated into recombinant α-carboxysomes (Figure 2a,b). In support of our observation, CbbQ was proposed to integrate into the carboxysome via interacting with the shell protein.^39^ It has been suggested that the Rca could be packed into β-carboxysomes and binds with Rubisco via its Rubisco small subunit-like domains and AAA+ core.^37^ As indicated by SDS-PAGE and immunoblot analysis, the Rubisco contents were similar among the four samples, suggesting that the integration of CbbO and CbbQ did not affect the Rubisco content (Figure 2a,b).

Negative-staining electron microscopy (EM) showed that the recombinant carboxysomes produced in the pS1D, pS1DO, pS1DQ, and pS1DQO constructs exhibited a polyhedral shape with defined edges and vertices (Figure 2c). The average diameters of the recombinant α-carboxysomes are 122 ± 19 nm for pS1D (mean ± SD, *n* = 43), 129 ± 14 nm for pS1DQ (*n* = 39), 124 ± 17 nm for pS1DO (*n* = 35), and 128 ± 18 nm for pS1DQO (*n* = 50) (Figure 2d). No significant difference in diameter was observed among the four types of recombinant carboxysomes, suggesting that integration of CbbO and CbbQ has no notable effects on the carboxysome structure. The sizes were comparable with those of the native carboxysome purified from *H. neapolitanus*(42) and the cyanobacterium *Synechococcus* WH8102,^43^ as well as recombinant *H. neapolitanus* carboxysomes^20^ and empty α-carboxysome shells produced in *E. coli*.^41^

### The Activase Activity of CbbO and CbbQ within the α-Carboxysome

**Figure 3:**
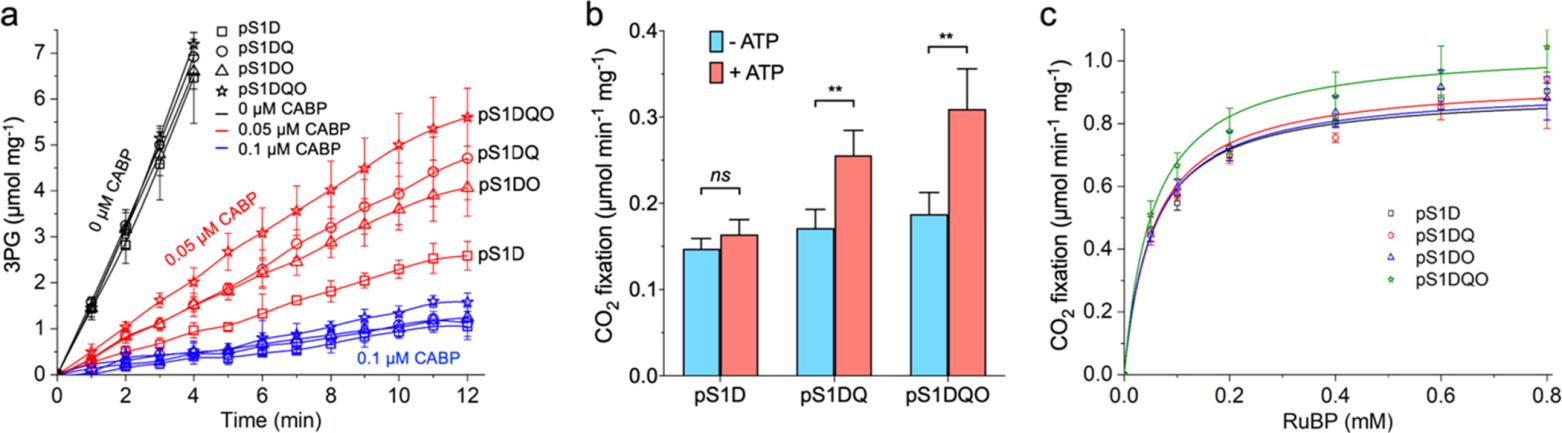
CbbQ and CbbO integrated into the α-carboxysomes function as a Rubisco activase to improve carboxylation. (a) CbbQ and CbbO function as Rca to elevate the tolerance of recombinant carboxysomes to CABP. Data show the rates of 3-phosphoglycerate (3PG) production from purified carboxysomes using an NADH (nicotinamide adenine dinucleotide hydrogen, reduced)-link coupling enzyme assay in the presence of CABP with varying concentrations. The measured Rubisco activities in the presence and absence of different concentrations of CABP are listed in Table 1. (b) ATP (adenosine triphosphate)-dependent Rca activities of CbbQ and CbbO. The measurement was conducted with the reaction buffer containing 0.05 μM CABP. *ns* (no significance), *p* > 0.05; **, *p* < 0.01. (c) Carbon fixation activities of isolated α-carboxysomes measured by ^14^C fixation, as a function of RuBP concentrations, fitted with the Michaelis–Menten equation. The analysis was carried out on the same sample presented in (a). The measured *V*_max_ and *K*_m_(RuBP) values are listed in Table 2. Error bars represent SD of at least three independent replicates.

While a functional CO_2_-concentrating mechanism (CCM) pathway has been reconstructed in *E. coli*,^26^ building on evidence that multiple proteins including CbbQO are required for CCM function, no study has yet examined the roles of CbbQO in isolated carboxysomes. Observing the successful incorporation of potentially functional activase proteins in recombinant carboxysomes, we examined the activase activity of CbbQO in these structures at different concentrations of carboxyarabinitol-1,5-bisphosphate (CABP), which is a tight-binding inhibitor of Rubisco.^44^ As expected, 0.1 μM CABP could inhibit up to 95% of Rubisco activity (Figure 3a; Table 1); Rubisco activity appeared to be linear in the absence of CABP, and there is no significant difference in the Rubisco activity between recombinant carboxysome types under these conditions. In contrast, remarkable differences were observed when assaying Rubisco activities at 0.05 μM CABP. The Rubisco activity of the pS1DQO carboxysomes was higher than that of pS1DQ (∼1.2 fold) and pS1DO (∼1.4 fold), and the pS1D carboxysomes that lack CbbQ and CbbO had the lowest Rubisco activity among these recombinant carboxysomes (Figure 3a, Table 1). Meanwhile, supplementing isolated carboxysomes with ATP could diminish Rubisco inhibition by CABP and enhance Rubisco activity (Figure 3b), consistent with the ATP requirement for CbbQ.^38^ Taken together, our results indicate that integration of both CbbQ and CbbO could improve the Rubisco carboxylation activities of recombinant carboxysomes, confirming their roles as Rca in dissociating the tightly bound CABP from the inhibited Rubisco holoenzymes and thereby enhancing the carboxylation of Rubisco.^38^ CbbQO has also been suggested to function as the Rca in both Form I and Form II Rubisco.^38,45^

**Table 1:** Rubisco Activities in the Presence and Absence of CABP at Different Concentrations in Isolated Recombinant α-Carboxysomes (*n* = 3)

	0 μM CABP	0.05 μM CABP	0.1 μM CABP
pS1D (nmol min^–1^ mg^–1^)	1708 ± 274	221 ± 32	82 ± 9
pS1DQ (nmol min^–1^ mg^–1^)	1896 ± 96	396 ± 49	85 ± 14
pS1DO (nmol min^–1^ mg^–1^)	1782 ± 63	358 ± 37	109 ± 10
pS1DQO (nmol min^–1^ mg^–1^)	2011 ± 65	515 ± 82	126 ± 23

To further evaluate the functions of CbbQ and CbbO in Rubisco activities of recombinant α-carboxysomes, we carried out ^14^C radiometric Rubisco assays as a function of the RuBP concentration (normalized by the total protein abundance) and then calculated *V*_max_ and *K*_m_ for RuBP using a Michaelis–Menten kinetic model. The pS1DQO carboxysomes possessed a higher *V*_max_ than the pS1D, pS1DQ, and pS1DO carboxysomes, indicating that the overall carbon-fixation activity of carboxysomes was stimulated in the presence of CbbQO (Figure 3c; Table 2). Moreover, immunoblot analysis indicated the equal quantities of Rubisco in these recombinant carboxysomes (Figure 2a,b), suggesting that the Rubisco functionality per active site was enhanced in the pS1DQO carboxysomes. *K*_m_(RuBP) of these recombinant carboxysomes was relatively similar (Table 2), suggesting that the CbbQO hetero-oligomer may specifically release tight-binding inhibitory sugar phosphates during Rubisco activation. Since incorporation of CbbQO could mediate activation of inhibited Rubisco and improve the CO_2_-fixation activities of carboxysomes (Figure 3), coexpressing the catalytically active CbbQO Rca and carboxysomes could be an effective approach to stimulate carboxysome function in heterologous hosts.^26^

**Table 2:** *V*_max_ and *K*_m_(RuBP) of Rubisco in Isolated Recombinant α-Carboxysomes (*n* = 3)

	pS1D	pS1DQ	pS1DO	pS1DQO
*V*_max_ (nmol min^–1^ mg^–1^)	961 ± 24	944 ± 39	972 ± 18	1067 ± 32
*K*_m_(RuBP) (μM)	68 ± 7	62 ± 5	62 ± 12	61 ± 9

### Variability of the α-Carboxysome Architecture

**Figure 4:**
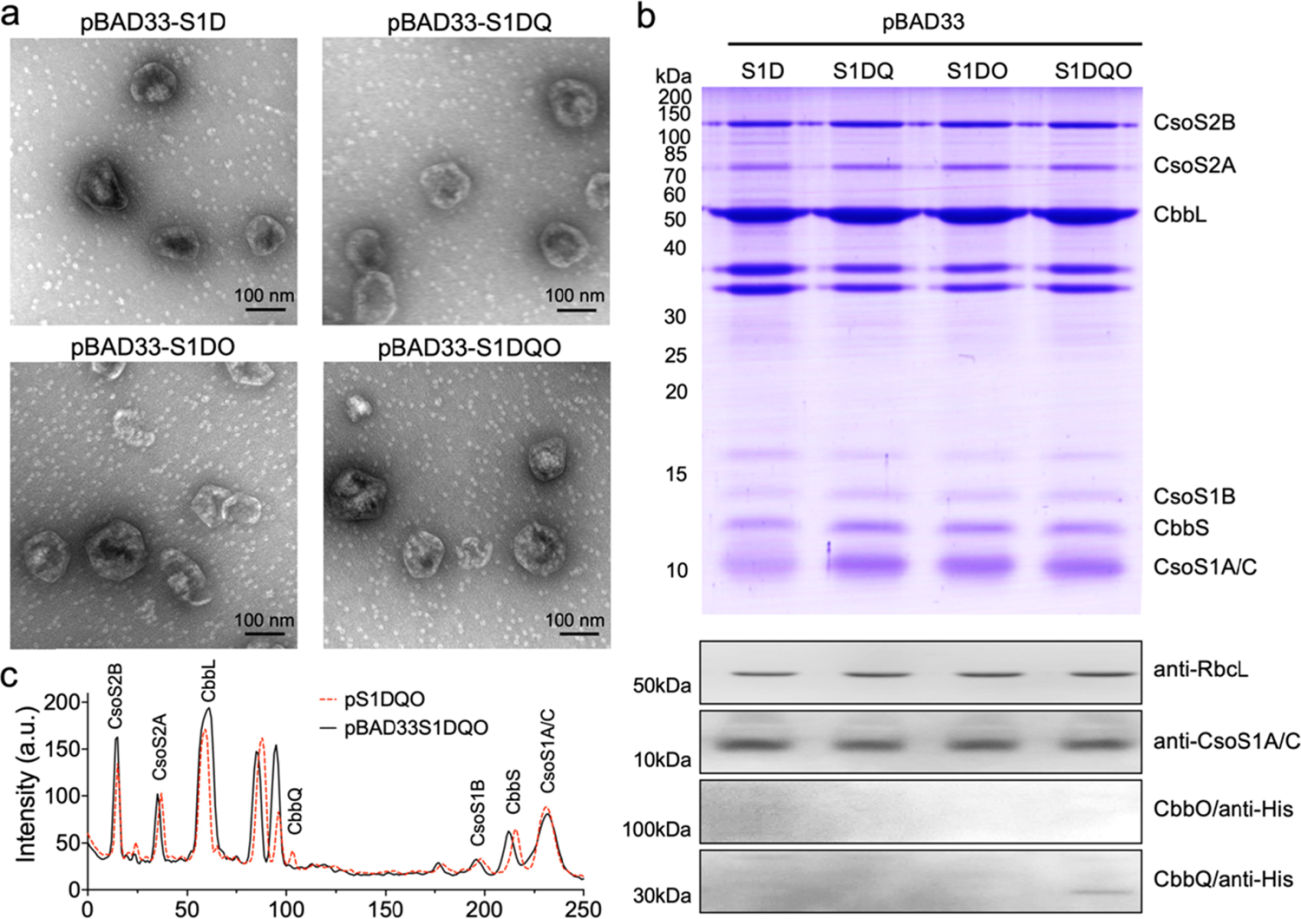
Analysis of recombinant α-carboxysomes produced by pBAD33. (a) EM images of isolated recombinant α-carboxysomes generated from the pBAD33 vectors. (b) SDS-PAGE (top) and immunoblot analysis (bottom) reveal the presence of major carboxysome proteins, including CsoS2A/B, CbbLS, and CsoS1A/B/C. In contrast to the pAM2991-expressing vectors, CbbO and CbbQ were not expressed or had low-level expression. The bands between 30 and 40 kDa are two membrane proteins from *E. coli*. (c) SDS-PAGE lane profile analysis of pS1DQO (Figure 2a) and pBAD33S1DQO (normalized by the CsoS1A/C content) shows the differences in the content of individual carboxysome components within the two types of recombinant α-carboxysomes. For example, notable changes were observed for CsoS2B, CbbL, and CbbQ.

To elucidate whether different expression systems can affect the formation and structure of carboxysomes, we also generated the carboxysome-expression vectors using a pBAD33 vector that is induced by arabinose. The carboxysome structure with a polyhedral shape could be formed by expressing the created pBAD33-S1D, pBAD33-S1DQ, pBAD33-S1DO, and pBAD33-S1DQO vectors (Figure 4a). No visible difference in size was observed between the recombinant carboxysomes expressed using different vectors. SDS-PAGE and immunoblot analysis showed the typical distribution pattern of α-carboxysome proteins (Figure 4b). However, the protein levels of CbbQ and CbbO were significantly lower in the purified recombinant carboxysomes expressed from the pBAD33-based vectors (Figure 4b) than those from the pAM2991 vector (Figure 2b). SDS-PAGE profile analysis further confirmed that the protein content of some components within the carboxysome structures differs among the carboxysomes generated by pAM2991 and pBAD33 vectors (Figure 4c). For example, the pBAD33-S1DQO carboxysome contains a relatively high content of Rubisco and CsoS2B in comparison with the pS1DQO carboxysomes, specifying the stoichiometric and organizational variations of the α-carboxysome architecture.

Stoichiometric plasticity has been recently assessed as a general feature of natural and recombinant BMCs, including β-carboxysomes,^13,14,46^ the propanediol utilization metabolosome,^47^ and several recombinant shell structures.^46,48,49^ This structural variation may have important implications on the flexible protein–protein interactions and the modulation of shell permeability for the regulation of BMC assembly and function in response to a varying environment. It also implies the requirement of tuning expression of carboxysome operons for functionality.^26^

## Conclusions

In this study, we experimentally verify that the Rca proteins CbbQ and CbbO could serve as structural components of reconstituted α-carboxysomes, without detectable effects on the carboxysome structure. Incorporation of both CbbQ and CbbO into the recombinant carboxysomes could promote catalytic activation of inhibited Rubisco in the presence of 0.05 μM CABP and enhance the CO_2_-fixation activities of recombinant carboxysomes in the presence of ATP. Moreover, we show that the assembly and organizational composition of recombinant carboxysomes could be modified by using different expression systems, highlighting the plasticity of the carboxysome architecture, which may be physiologically vital for carboxysome self-assembly, repair, and permeability regulation. Our study may offer new strategies for rational design, engineering, and modulation of carboxysome structure and function in synthetic biology, emphasizing the requirement for carboxysomal Rca for correct functions.

## Methods

### Construction of Expressing Vectors

The genetic organization of the operons that express α-carboxysomes is displayed in Figure 1c. For pS1D, the operon was amplified from the pHnCBS1D plasmid (Addgene plasmid # 52065)^20^ and then cloned into a modified pAM2991 vector containing a Kanamycin resistance gene by Gibson Assembly (NEB, UK). The *cbbQ* and *cbbO* genes were cloned from the genomic DNA (deoxyribonucleic acid) of *H. neapolitanus*, and a His-tag coding sequence was appended to the 3′-termini of *cbbQ* and *cbbO* by PCR. The fragments of *cbbQ* and *cbbO* were digested by *Bsa*I and then assembled by T_4_ DNA ligase using Golden Gate Assembly^50^ to generate the *cbbQO* expression cassette. Finally, *cbbQ*, *cbbO*, and *cbbQO* were cloned into the pS1D vector at the *Not*I site to generate pS1DQ, pS1DO, and pS1DQO, respectively. To generate the pBAD33-S1D, pBAD33-S1DQ, pBAD33-S1DO, and pBAD33-S1DQO vectors, the operons in pS1D, pS1DQ, pS1DO, and pS1DQO were cloned into the amplicon of a pBAD33 vector^51^ by Gibson Assembly. The positive clones were verified by PCR, and the plasmids were finally confirmed by sequencing. All the primer information used in this research is listed in Table 3.

**Table 3:** Primers Used in This Study^a^

primer	sequence
pS1D-F	**cacaggaaacagaccatggaattc**atggcagttaaaaagtatagtgctggtg
pS1D-R	**ctgcaggtcgactctagaggatcc**gattactttctgttcgacttaagcattatggc*gcggccgc*ttagaacccttca
CbbQ-F	**cgcgctgaagggttctaa**cgaaatacaaggcaatttaaatg
CbbQ-R	**ctgttcgacttaagcattatgc***ggtctc*gtacattagtgatggtgatggtgatgaaagaacgttttgacgacgg
CbbO-F	**cgcgctgaagggttctaac***ggtctc*gtgtatggccagattgattttgtccg
CbbO-R	**ctgttcgacttaagcattatgc**ttagtgatggtgatggtgatgtcgcgtcatcgacaaataaagtg
pBAD33S1D-F	**gtttaactttaagaaggagatataca**atggcagttaaaaagtatagtgctggtg
pBAD33S1DQO-R	**ctacgcctgaataagtgc**tgcaggcggccctgttcgacttaagcattatg
pBAD33-R	**tgtatatctccttcttaaagttaaacaaaattatttctagagg**
pBAD33-F	**gcacttattcaggcgtagcaac**

a Homologous sequences for Gibson assembly, restriction enzyme sites, and His-tag coding sequences are shown in bold, italic, and underlined, respectively.

The vector construction was carried out in *E. coli* strain BL21(DE3)/TOP10 at 37 °C in the lysogeny broth (LB) medium with 10 μg mL^–1^ chloramphenicol or 50 μg mL^–1^ kanamycin.

### Protein Expression and Carboxysome Purification

The *E. coli* BL21(DE3)/TOP10 constructs were cultured overnight at 37 °C in 10 mL of LB medium with the corresponding antibiotic, and the cultures were diluted in 800 mL of medium in a 2-L flask. When the optical density (OD) of the culture reaches 0.6, arabinose or IPTG was added to a final concentration of 1 mM or 50 μM to induce protein expression. The cultures were grown at 25 °C overnight with a 120-rpm shaking.

Cells were harvested by centrifugation at 5000*g* for 10 min and washed with 10 mL of TEMB buffer (10 mM Tris-pH 8.0, 10 mM MgCl_2_, 1 mM EDTA (ethylenediamine tetraacetic acid), and 20 mM NaHCO_3_). The cells were then resuspended in 20 mL of TEMB buffer with the 10% CelLytic B cell lysis reagent (Sigma-Aldrich, USA) and 1% Protease Inhibitor Cocktail (Melford, UK). The cells were broken by sonication and then centrifuged at 10,000*g* to remove cell debris at 4 °C. The supernatant was recentrifuged at 50,000*g* for 30 min at 4 °C to enrich carboxysomes. The pellet was resuspended with 2 mL of TEMB buffer and centrifuged at 10,000*g* for 1 min, before loading the supernatant onto a 10, 20, 30, 40, and 50% (w/v) sucrose density gradient. Sucrose gradients were subjected to centrifugation at 80,000*g* for 30 min at 4 °C. Carboxysomes were enriched in the 40% sucrose fraction and were collected for further analysis.

### SDS-PAGE and Immunoblot Analysis

SDS-PAGE and immunoblot analysis were performed as described previously.^23,31,52^ Protein concentrations were quantified by the Bradford method.^53^ Anti-RbcL (1:10,000 dilution, Agrisera, Sweden), anti-CsoS1 from *H. neapolitanus* (1:5000 dilution, Agrisera, Sweden), and anti-HisTag (Invitrogen, USA) antibodies and horseradish peroxidase-conjugated goat antirabbit immunoglobulin G secondary antibody were used for immunoblot analysis and imaged on an Image Quant LAS 4000 platform (GE Healthcare Life Sciences, USA).

### Rubisco Activity and Activase Assays

Rubisco activity assays were performed as previously described.^13^ Approximately 200 ng μL^–1^ isolated α-carboxysomes (5 μL) in Rubisco assay buffer (100 mM EPPS (4-(2-hydroxyethyl)-1-piperazinepropanesulphonic acid), pH 8.0, 20 mM MgCl_2_, 3.5 mM ATP) were aliquoted into scintillation vials containing NaH^14^CO_3_ (1.48–2.22 GBq mmol^–1^) at a final concentration of 25 mM and incubated at 30 °C for 2 min. d-Ribulose-1,5-bisphosphate sodium salt hydrate (RuBP; Sigma-Aldrich) was then added to the samples with a range of concentrations (0–0.8 mM) to initiate carbon fixation. The reaction was terminated after 5 min incubation by adding 10% (v/v) formic acid. The samples were then dried on heat blocks at 95 °C to remove unfixed NaH^14^CO_3_, and the pellets were resuspended in distilled water in the presence of the scintillation cocktail (Ultima Gold XR; Perkin-Elmer, USA). Radioactivity measurements were carried out using a scintillation counter (Tri-Carb; Perkin-Elmer, USA). Counts per minute were used to calculate the amount of fixed ^14^C according to the standard curve and were then converted to the total CO_2_ fixation rates. *V*_max_ was calculated using a Michaelis–Menten kinetic model in Origin Pro 2020b (OriginLab, USA). For each experiment, at least three independently purified carboxysome samples were examined. Results are presented as mean ± SD.

For Rubisco activase activity analysis, 1 μg of purified carboxysome was preincubated with 100 μL of prereaction buffer (100 mM EPPS, pH 8.2, 20 mM MgCl_2_, 1 mM EDTA, 3.5 mM ATP, 5 mM phosphocreatine, 0.25 mM NADH, 25 mM bicarbonate, 5 U mL^–1^ creatine phosphokinase, 5 U mL^–1^ 3-phosphoglycerate kinase, 5 U mL^–1^ NAD-dependent glyceraldehyde 3-phosphate dehydrogenase, and CABP) at 30 °C for 10 min in the 96-well plates. The reaction was started by adding 100 μL of reaction buffer (100 mM EPPS, pH 8.2, 20 mM MgCl_2_, 1 mM EDTA, 3.5 mM ATP, 5 mM phosphocreatine, 0.25 mM NADH, 25 mM bicarbonate, 1 mM RuBP (final concentration: 0.5 mM), 5 U mL^–1^ creatine phosphokinase, 5 U mL^–1^ 3-phosphoglycerate kinase, 5 U mL^–1^ NAD-dependent glyceraldehyde 3-phosphate dehydrogenase, and CABP) at 30 °C, and the concentration of NADH was continually tracked by the absorption of 340 nm for every minute. The NADH oxidation rate was converted to the 3PG rate to represent the carbon fixation efficiency.^20^

The ATP-dependent assay was carried out using the radioactivity assay as described above. In detail, 1 μg of purified carboxysome was preincubated with 235 μL of prereaction buffer (±3.5 mM ATP) containing 0.05 μM CABP at 30 °C for 5 min, and RuBP was then added to 1 mM to initiate the reaction.

### Electron Microscopy

The structures of purified recombinant α-carboxysomes were characterized using negative-staining transmission electron microscopy as described previously.^13,23,31^ The sizes of the recombinant carboxysomes were analyzed by ImageJ.

## Abbreviations

BMC: bacterial microcompartment
CA: carbonic anhydrase
CABP: carboxyarabinitol-1,5-bisphosphate
CBB: Calvin–Benson–Bassham
PDU: propanediol utilization
Rca: Rubisco activases
Rubisco: ribulose-1,5-bisphosphate carboxylase oxygenase
RuBP: ribulose-1,5-bisphosphate
SSUL: small subunit-like
3PG: 3-phosphoglycerate
